# Association of SARC-F with muscle mass, strength, and quality derived from bioelectrical impedance spectroscopy for sarcopenia assessment

**DOI:** 10.1038/s41598-025-25862-z

**Published:** 2025-11-25

**Authors:** Tsukasa Yoshida, Yuki Nishida, Emi Kondo, Miwa Yamaguchi, Aya Itoi, Hiroyuki Shirato, Hirohiko Hirano, Satoshi Sasaki, Minoru Yamada, Shigeho Tanaka, Misaka Kimura, Fuminori Katsukawa, Yosuke Yamada

**Affiliations:** 1 National Institute of Health and Nutrition, National Institutes of Biomedical Innovation, Health and Nutrition, Tokyo, Japan; 2 Institute for Active Health, Kyoto University of Advanced Science, Kyoto, Japan; 3 Sports Medicine Research Center, Keio University, Kanagawa, Japan; 4 Faculty of Health and Sport Sciences, University of Tsukuba, Tsukuba, Japan; 5 Japan Society for the Promotion of Science, Tokyo, Japan; 6 Faculty of Health and Welfare, Kobe Women’s University, Hyogo, Japan; 7 Silverpia-Kaga Long-Term Care Health Facility, Tokyo, Japan; 8 Dentistry and Oral Surgery, Tokyo Metropolitan Institute of Gerontology, Tokyo, Japan; 9 Department of Social and Preventive Epidemiology, Graduate School of Medicine, The University of Tokyo, Tokyo, Japan; 10 Faculty of Human Sciences, University of Tsukuba, Tsukuba, Japan; 11 Faculty of Nutrition, Kagawa Nutrition University, Saitama, Japan; 12 Department of Medicine and Science in Sports and Exercise, Graduate School of Medicine, Tohoku University, Miyagi, Japan; 13 Department of Sports and Health Sciences, Graduate School of Biomedical Engineering, Tohoku University, Miyagi, Japan; 14 Department of Sport Sciences, Osaka University of Health and Sport Sciences, Osaka, Japan; 15 College of Gastronomy Management, Ritsumeikan University, Shiga, Japan

**Keywords:** Bioelectrical impedance spectroscopy, Muscle quality, Phase angle, SARC-F, Sarcopenia, Geriatrics, Nutrition, Ageing, Musculoskeletal system

## Abstract

The SARC-F is a 5-item questionnaire screening for patients at risk of sarcopenia. The electrical properties of tissues derived from bioelectrical impedance spectroscopy (BIS), including phase angle (PhA) and intracellular-extracellular water ratio (ICW/ECW) indices, are biomarkers of age-related loss of skeletal muscle quantity and quality. The purpose of the current study was to examine the association between SARC-F and muscle mass, strength, muscle-specific strength, and muscle quality as assessed by BIS and hand grip strength (HGS). Sarcopenia risk was assessed using the SARC-F questionnaire. BIS was used to obtain skeletal muscle mass (SMM) and skeletal muscle mass index (SMI), PhA, ICW/ECW index, membrane capacitance (Cm) and characteristic frequency (fc) for assessing muscle quantity and quality. HGS was evaluated, and muscle-specific strength (HGS/SMM) was calculated. The Pearson correlation coefficient was used to examine the associations between SARC-F scores and each muscle parameter. A total of 205 older adults aged 65–99 years were included in the current cross-sectional analysis. Of those, 142 were community-dwelling healthy older adults (110 women and32 men) and 63 were the older adults living in special nursing homes for the older adults (45 women and 18 men). SARC-F was significantly correlated with SMM, SMI, HGS, and HGS/SMM in both men and women (*p* < 0.05). SARC-F was also correlated with PhA, ICW/ECW index, Cm, and fc in both men and women (*p* < 0.001). The SARC-F score was associated with SMM, HGS, HGS/SMM, PhA, ICW/ECW index, Cm, and fc independently of age, sex, height, and BMI. SARC-F is a subjective yet simple assessment tool for muscle mass, strength, and quality relevant to sarcopenia.

## Introduction

In 1989, sarcopenia was originally defined by Rosenberg as an age-related loss of skeletal muscle mass (SMM)^1^. In 2010, the European Working Group on Sarcopenia in Older People (EWGSOP) defined sarcopenia as the presence of low muscle mass associated with loss of strength and/or low physical performance^2^. The definition was recently updated by the same group (EWGSOP2)^3^, stating that the isolated finding of low muscle strength is sufficient to define the condition as “probable sarcopenia,” and when accompanied by low muscle mass or muscle quality, the diagnosis of sarcopenia can be confirmed. According to the EWGSOP2 consensus, physical performance is no longer regarded as a diagnostic criterion but a factor related to disease severity.

The importance of early detection of sarcopenia in older adults has been proposed recently^3–5^. The EWGSOP2, International Clinical Practice Guidelines for Sarcopenia (ICFSR), Society on Sarcopenia, Cachexia and Wasting Disorders (SCWD) position paper, and the Asian Working Group for Sarcopenia (AWGS) 2019 emphasize that screening is the first step in identifying individuals with sarcopenia^3–6^. The use of the SARC-F questionnaire is recommended by the EWGSOP2, ICFSR, SCWD, and AWGS 2019^3–6^.

The SARC-F is a unique questionnaire developed for screening patients at risk of sarcopenia^7–9^. The SARC-F comprises five items: strength, assistance in walking, rise from a chair, climb stairs, and falls, with a 3-level score (range 0 to 2 points) for each item; representing none (0), some (1), or a lot (2) (with the exception of falls, which is evaluated as none [0], 1–3 times^1^, or ≥ 4 times^2^). The total score ranges from 0 to 10, with scores ≥ 4 considered a criterion for sarcopenia^8^. The SARC-F score had a similar performance in predicting the incident physical limitation and mortality with regard to the actual sarcopenia definitions^10–16^. A scoping review summarizing all validated tools for screening sarcopenia showed that the SARC-F questionnaire was the most commonly used across various settings, including community-dwelling populations, hospitals, nursing homes, and health checkups^17^. Although it is characterized by low sensitivity and high specificity^18^, its practicality makes it a commonly used first-step screening tool in clinical and community settings.

It has traditionally been hypothesized that the age-related reduction in muscle strength results mainly from the loss of SMM. However, the age-related decrease in SMM in insufficient to explain the decline in muscle strength^19,20^. Skeletal muscle tissue includes extracellular tissue and bundles of metabolically heterogeneous fiber types^21,22^. Fibers atrophy with age, and the level of fibrosis and intramuscular fat increase within and around the bundles^23,24^. This age-related increase in the non-contractile content of muscle is related to the loss of muscle strength^25^. Bioelectrical impedance spectroscopy (BIS) can be used to distinguish intracellular water (ICW) from extracellular water (ECW)^26^ and to evaluate muscle quality^21,27–29^. Previous studies indicated that the electrical properties of tissues assessed by BIS, like intracellular-to-extracellular water (ICW/ECW) index, phase angle (PhA), membrane capacitance (Cm), and characteristic frequency (fc), are reasonable metrics to estimate functional muscle mass and muscle quality^21,30^. Many studies have investigated the relationship between PhA and sarcopenia in various older populations, including community-dwelling individuals, and patients with cancer, cardiovascular and cerebrovascular diseases, kidney disease, chronic obstructive pulmonary disease, and Parkinson’s disease^31,32^. A recent meta-analysis showed that although underlying health conditions may influence this association, PhA remains a potentially robust screening tool for sarcopenia^33^. Moreover, BIS could provide insights into inter-individual variance in maximum muscle strength, which cannot be explained by the dual-energy X-ray absorptiometry (DXA)-measured lean mass^21,34,35^.

We hypothesized that the SARC-F score was associated with muscle quality, as well as muscle quantity and strength. The purpose of the current study was to examine the association between the SARC-F score and BIS-derived muscle quantity and quality in older adults. In particular, we aimed to clarify the relationship between SARC-F score and the electrical properties of tissues derived from BIS.

## Materials and methods

This was the cross-sectional study. Community-dwelling healthy adults were voluntarily recruited from 406 participants of an ongoing annual physical function checkup study conducted at Kyoto University of Advanced Science^36,37^. Inclusion criteria for community-dwelling adults were as follows: (a) reported ability to walk more than 10 m with or without a cane, (b) ability to provide informed consent with no indication of dementia, (c) no history of any joint arthroplasty or current use of an artificial pacemaker, (d) no current medication for edema or lymphedema, and (e) absence of any definitive kidney, digestive, or other acute diseases. The older adults living in special nursing homes were recruited from five facilities in Tokyo^38,39^. Subjects were excluded if they had infectious or serious diseases, used medication that could affect energy or water metabolism, or were at risk of aspiration^38,39^. All participants were fully informed of the purpose, procedures, and risks of the study and provided written informed consent before participation. In older residents of the facility, we recruited those who were judged to have no cognitive impairment based on previous medical records and who were capable of providing informed consent independently. The study protocol was approved by the Ethics Committees of Keio University (No. 2015-03), National Institutes of Biomedical Innovation, Health and Nutrition (No. NIBIOHN29), and Kyoto University of Advanced Sciences (No. 27-2), and the study was conducted in compliance with global, national, and local regulations. The study period was between June 2016 and April 2017. Indicators such as BIS, SARC-F, and HGS were collected on the same day.

### Bioelectrical impedancespectroscopy

The basic theory of BIS has been described elsewhere^21,26,27^. Briefly, bioelectrical impedance was measured using a logarithmic distribution of 256 frequencies ranging from 4 to 1,000 kHz (SFB7, ImpediMed, Pinkenba, QLD, Australia), using disposable tab-type monitoring electrodes (2 cm × 2 cm, Medtronic, Minneapolis, MN). The R_0_ and R_∞_ for the whole body were determined by extrapolation after fitting the spectrum of bioimpedance data to the Cole–Cole model (Bioimp software, ImpediMed). For the BIS, the R_ECW_ was equal to R_0_, and the R_ICW_ was calculated using the formula 1/[(1/R_∞_) − (1/R_0_)]. The intracellular impedance index was calculated as height^2^/R_ICW_, and the extracellular resistance index was calculated as height^2^/R_ECW_. Height^2^/R_ICW_ reflects ICW, while, muscle cell mass (MCM)^20,40,41^ and height^2^/R_ECW_ reflect ECW^42,43^. The resistance ratio, calculated as R_ECW_/R_ICW_, is an index of the ratio of ICW to ECW (ICW/ECW). The Cm, fc, and PhA were also obtained from the Cole–Cole model. The SM was calculated using the bioelectrical impedance analysis (BIA) equation proposed by Janssen et al.^44^: SM (kg) = [(height^2^/R_50_ × 0.401) + (sex × 3.825) + (age ×  − 0.071)] + 5.102, where height is measured in centimeters; R_50_ is measured in ohms between the right wrist and ankle in a supine position; sex is recorded as: men = 1 and women = 0; and age is measured in years. This BIA equation was developed against magnetic resonance imaging (MRI) measures of whole-body muscle volume in a sample of 269 men and women who varied widely in age (18–86 years) and adiposity [body mass index (BMI), 16–48 kg/m^2^]. MRI being a measure of muscle volume, in that study, the volume units were converted to mass units by multiplying the volumes by the assumed constant density of adipose tissue-free SM (1.04 kg/l). In that study, the correlation between BIA-predicted and MRI-measured SMM was 0.93, with an SE of 9%^44^. Absolute SMM (kg) was normalized for squared height [SM (kg)/height^2^ (m^2^)] and was termed the skeletal muscle index (SMI). The cutoff point for severe sarcopenia of SMI was set at 8.50 kg/m^2^ for men and 5.75 kg/m^2^ for women^2^.

### Hand grip strength

Hand grip strength (HGS) tests were conducted after the BIS measurements. Maximal HGS was measured using a Smedley hand dynamometer (Grip-D, TKK5401; Takei Scientific Instruments, Niigata, Japan), as described elsewhere^36^. The involved arm was placed in complete extension with the dynamometer not touching any other part of the body except for the hand being measured. Two trials were allowed for each hand alternately, separated by a brief rest, and the highest value was recorded. The participants were encouraged to exert themselves maximally during each effort. The average of the maximum HGS recordings on each side was used. The cutoff point for weakness of HGS was set at 30 kg for men and 20 kg for women, in accordance with the data provided by previous studies^2,45,46^. HGS was divided by SMM (HGS/SMM) in order to calculate another muscle quality index.

### SARC-F questionnaire

The SARC-F is a questionnaire designed for screening sarcopenia in older adults. It addresses the following five domains: strength, assistance in walking, rise from a chair, climb stairs, and falls^7,8^. Each domain has one question, and the self-reported answer is scored from 0 to 2 points per domain. The Japanese version of the SARC-F questionnaire, which was developed in accordance with the WHO translation guidelines by experts in sarcopenia research, was used^47,48^. Yosuke Yamada first translated the original English version into Japanese, Minoru Yamada examined the preliminary version, and modified it wherever necessary. A Japanese person fluent in English back-translated it into English, and the English version was checked by a native English speaker. This process was repeated until the back-translated English version became equivalent to the original version^47,48^. The SARC-F questionnaire was administered through face-to-face interviews conducted by trained research staff. In cases where participants had difficulty writing due to physical limitations, such as hand paralysis, staff assisted by recording their verbal responses.

### ADL, frailty, and appetite in the older adults living in special nursing home

We assessed the Barthel index (0 to 100) for evaluating their activities of daily living (ADL), the FRAIL score for evaluating their frailty (0 to 5), and SNAQ (Simplified Nutritional Appetite Questionnaire) for evaluating their appetite (4 to 20) in the older adults living in a special nursing home to describe the characteristics of those participants.

### Statistical analyses

To examine if the SARC-F score is a significant predictor of muscle quantity or quality, the sample size was calculated using multiple linear regression analysis with seven predictors with an effect size *f*^2^ of 0.1, α of 0.01, and power of 0.95, which amounted to a sample size of 182 (G*Power 3.1.9.7, Universität Kiel, Germany). The normality of the variables was tested with the Kolmogorov–Smirnov test. Results were presented as the mean ± SD or median [25th and 75th percentiles]. The variables between men and women were compared using analysis of variance. Pearson’s correlation coefficients (r) were calculated to examine the relationship between SARC-F and SMM, SMI, MCM index, ICW/ECW index, PhA, Cm, fc, HGS, or HGS/SMM. In the linear model, age, sex, height, BMI, HGS, and the SARC-F scores were entered as independent variables. Receiver Operating Characteristic (ROC) curve analyses were performed to evaluate the diagnostic performance of the SARC-F in identifying sarcopenia. Two reference standards were applied for sarcopenia diagnosis: SMI derived from BIS and handgrip strength. The area under the ROC curve (AUC) with 95% confidence intervals (CI) was calculated to quantify the accuracy of the SARC-F in discriminating sarcopenia cases. Analyses were performed using SPSS software (Ver. 22.0, IBM Corp., Armonk, NY, USA), and R software (Ver. 4.5.0; R Foundation for Statistical Computing, Vienna, Austria). ROC curve analyses were conducted using the pROC package to evaluate the diagnostic performance of the SARC-F. Statistical significance was set at *p* < 0.05.

## Results

A total of 205 older adults (range, 65–99 years) were recruited for this study. Of these, 142 were community-dwelling healthy older adults (110 women and 32 men) and 63 were living in long-term care facilities for older adults (45 women and 18 men). General characteristics of the participants are presented in Table 1. Men had significantly higher values of height, weight, BMI, grip strength, SMM, and SMI than women. For BIS measurements, men had significantly higher values of ICW index, ICW/ECW index, Cm, and PhA, while lower values of fc and resistance at 50 kHz than in women. The mean and SD of the Barthel index, FRAIL score, and SNAQ in the older adults living in special nursing homes were 67 ± 20, 1.68 ± 1.19, and 15.3 ± 1.9, respectively.

**Table 1:** General characteristics of study participants.

	Women		Men		*p* Value
	Mean	SD	Mean	SD	
Age (y)	77.2	8.0	78.3	6.4	0.371
Height (cm)	150.4	6.2	163.4	6.6	< 0.001
Weight (kg)	48.7	7.8	57.6	8.7	< 0.001
BMI (kg/m^2^)	21.5	2.6	21.6	3.1	0.739
Grip Strength (kg)	18.6	6.4	28.2	9.5	< 0.001
SMM (kg)	15.7	3.0	25.3	3.8	< 0.001
SMI (kg/m^2^)	6.9	1.0	9.4	1.2	< 0.001
ICW/ECW index	0.31	0.08	0.33	0.08	0.036
C_m_ (nF)	1.0	0.4	1.4	0.6	< 0.001
fc (kHz)	59.2	14.3	55.7	15.0	0.139
R at 50 kHz (Ohm)	578.2	74.0	498.3	67.6	< 0.001
Phase angle (degrees)	4.4	1.1	4.8	1.1	0.020

BMI, body mass index; SMM, skeletal muscle mass; SMI, skeletal muscle mass index; ICW, intracellular water; Cm, membrane capacitance; fc, characteristic frequency; R, resistance. P values show statistical differences between women and men.

**Fig. 1:**
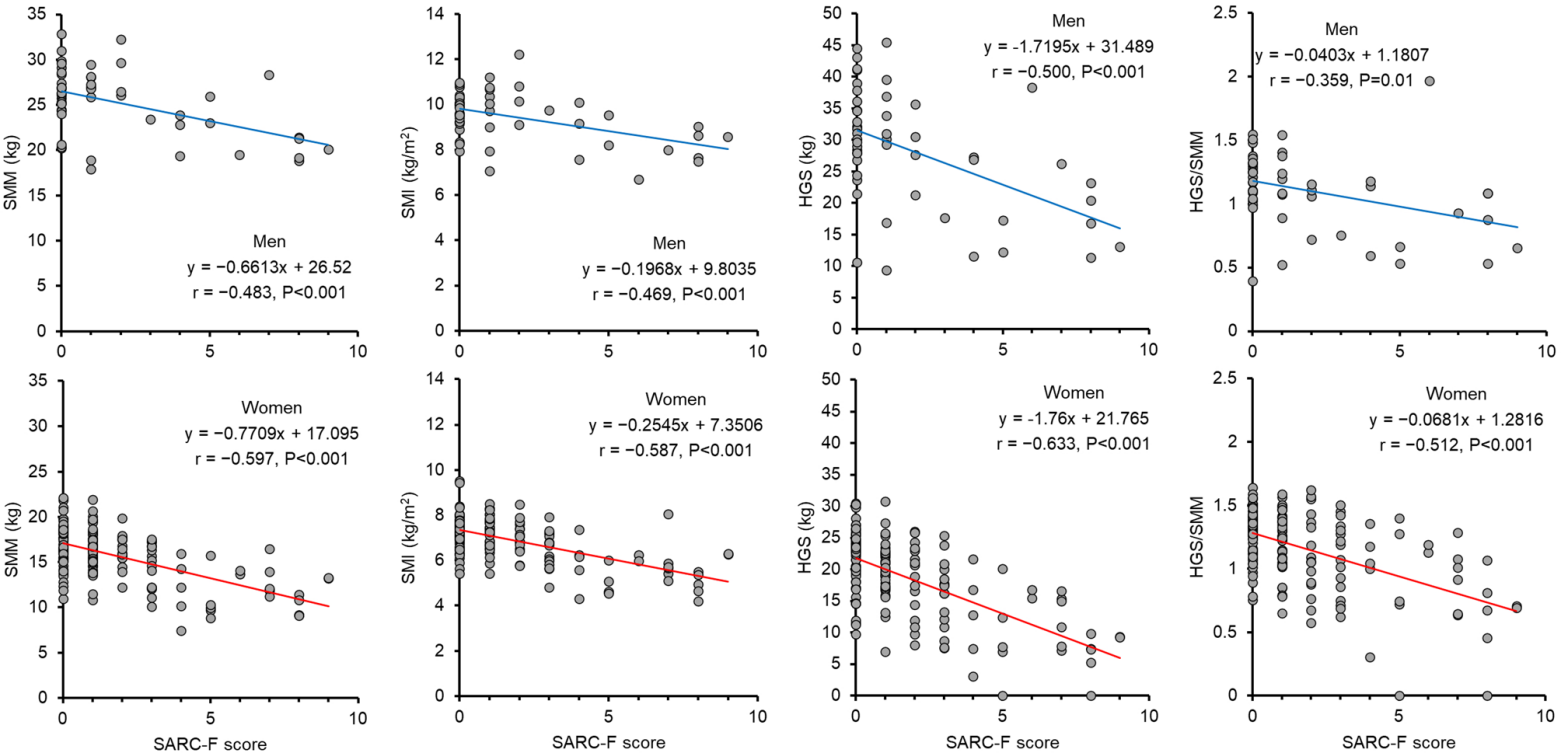
Relationship between the SARC-F score and skeletal muscle mass (SMM), skeletal muscle mass index (SMI), hand grip strength (HGS), or the ratio of HGS/SMM in men and women.

**Fig. 2:**
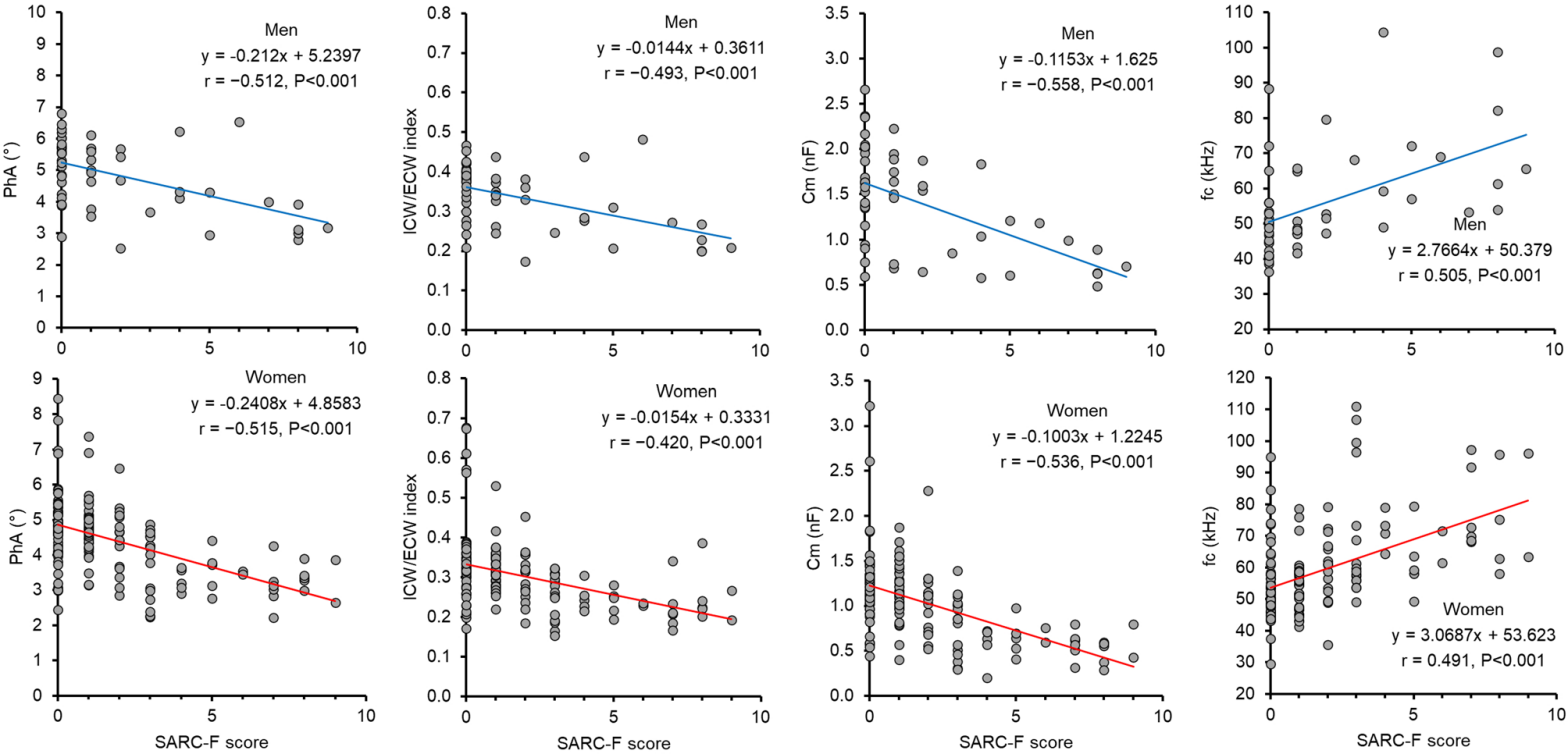
Relationship between the SARC-F score and phase angle (PhA), the intracellular-to-extracellular water (ICW/ECW) index, membrane capacitance (Cm), and characteristic frequency (fc) in men and women.

We examined the relationship between the SARC-F score and SMM, SMI, HGS and HGS/SMM (Figure 1). The SARC-F score was significantly correlated with SMM, SMI, HGS, and HGS/SMM in both men and women (*p* < 0.05). Subsequently, we investigated whether the SARC-F score would be informative in predicting muscle quality derived from BIS measures or muscle strength tests, as shown in Figure 2. The SARC-F score was significantly correlated with PhA, ICW/ECW index, Cm, and fc obtained from the BIS both men and women (*p* < 0.001).

The results of the multiple linear regression analysis for muscle mass and quality are presented in Tables 2 and 3. The SARC-F score was significantly associated with SMM, HGS, and HGS/SMM independently of age, sex, height, and BMI (*p* < 0.001). The SARC-F score was also significantly associated with PhA, ICW/ECW index, Cm, and fc independently of age, sex, height, BMI, and SMM (*p* < 0.05).

**Table 2:** Multiple linear regression analyses for skeletal muscle mass, strength or muscle quality.

	Unstandardized		Standard	
	B	(95% CI)	β	*p* value
a. Dependent variable: Skeletal muscle mass (SMM)				
Adj. R^2^ = 0.899				
(Constant)	− 25.292	(− 33.874, − 16.71)		< 0.001
Age (y)	− 0.068	(− 0.106, − 0.031)	− 0.101	< 0.001
Sex (men = 1, women = 0)	6.319	(5.538, 7.101)	0.525	< 0.001
Height (cm)	0.254	(0.211, 0.297)	0.409	< 0.001
BMI (kg/m^2^)	0.397	(0.309, 0.485)	0.211	< 0.001
SARC-F score	− 0.285	(− 0.395, − 0.176)	− 0.132	< 0.001
b. Dependent variable: Hand grip strength (HGS)				
Adj. R^2^ = 0.683				
(Constant)	− 10.712	(− 35.085, 13.662)		0.387
Age (y)	− 0.312	(− 0.418, − 0.206)	− 0.286	< 0.001
Sex (men = 1, women = 0)	6.234	(4.014, 8.453)	0.323	< 0.001
Height (cm)	0.291	(0.169, 0.412)	0.291	< 0.001
BMI (kg/m^2^)	0.522	(0.271, 0.773)	0.173	< 0.001
SARC-F score	− 0.875	(− 1.186, − 0.564)	− 0.253	< 0.001
c. Dependent variable: HGS/SMM				
Adj. R^2^ = 0.359				
Constant	2.188	(0.896, 3.480)		0.001
Age (y)	− 0.016	(− 0.021, − 0.010)	− 0.393	< 0.001
Sex (men = 1, women = 0)	− 0.047	(− 0.165, 0.071)	− 0.066	0.432
Height (cm)	0.001	(− 0.005, 0.007)	0.027	0.762
BMI (kg/m^2^)	0.005	(− 0.009, 0.018)	0.041	0.500
SARC-F score	− 0.035	(− 0.051, − 0.018)	− 0.272	< 0.001

BMI, body mass index; SMI, skeletal muscle mass index; HGS, hand grip strength; SMM, skeletal muscle mass.

**Table 3:** Multiple linear regression analyses for muscle quality evaluated by electrical properties of BIS.

	Unstandardized		Standard	
	B	(95% CI)	β	*p* value
a. Dependent variable: ICW/ECW index				
Adj. R^2^ = 0.347				
(Constant)	0.467	(0.085, 0.848)		0.017
Age (y)	− 0.003	(− 0.005, − 0.002)	− 0.304	< 0.001
Sex (men = 1, women = 0)	− 0.020	(− 0.069, 0.028)	− 0.103	0.416
Height (cm)	0.000	(− 0.002, 0.002)	− 0.006	0.959
BMI (kg/m^2^)	0.001	(− 0.003, 0.006)	0.043	0.548
SMM (kg)	0.006	(0.000, 0.011)	0.346	0.056
SARC-F score	− 0.006	(− 0.011, − 0.001)	− 0.166	0.018
b. Dependent variable: Phase angle (PhA)				
Adj. R^2^ = 0.499				
(Constant)	7.484	(3.102, 11.867)		< 0.001
Age (y)	− 0.055	(− 0.073, − 0.036)	− 0.379	< 0.001
Sex (men = 1, women = 0)	− 0.245	(− 0.802, 0.312)	− 0.096	0.387
Height (cm)	− 0.005	(− 0.031, 0.022)	− 0.036	0.726
BMI (kg/m^2^)	0.034	(− 0.016, 0.083)	0.084	0.180
SMM (kg)	0.082	(0.016, 0.148)	0.387	0.015
SARC-F score	− 0.086	(− 0.141, − 0.031)	− 0.188	0.002
c. Dependent variable: Membrane capacitance (Cm)				
Adj. R^2^ = 0.647				
(Constant)	2.112	(0.468, 3.756)		0.012
Age (y)	− 0.012	(− 0.019, − 0.005)	− 0.185	< 0.001
Sex (men = 1, women = 0)	− 0.283	(− 0.492, − 0.075)	− 0.249	0.008
Height (cm)	− 0.013	(− 0.023, − 0.003)	− 0.218	0.011
BMI (kg/m^2^)	0.022	(0.004, 0.041)	0.125	0.019
SMM (kg)	0.086	(0.061, 0.111)	0.913	< 0.001
SARC-F score	− 0.028	(− 0.048, − 0.007)	− 0.136	0.009
d. Dependent variable: Characteristics frequency (fc)				
Adj. R^2^ = 0.480				
(Constant)	53.431	(− 5.432, 112.293)		0.075
Age (y)	0.742	(0.494, 0.991)	0.382	< 0.001
Sex (men = 1, women = 0)	0.143	(− 7.301, 7.588)	0.004	0.970
Height (cm)	− 0.145	(− 0.497, 0.207)	− 0.083	0.418
BMI (kg/m^2^)	− 1.257	(− 1.920, − 0.594)	− 0.239	< 0.001
SMM (kg)	-0.295	(− 1.183, 0.592)	− 0.105	0.512
SARC-F score	1.207	(0.460, 1.955)	0.200	0.002

BMI, body mass index; SMM, skeletal muscle mass; ICW, intracellular water; ECW, extracellular water; PhA, phase angle; Cm, membrane capacitance; fc, characteristic frequency.

**Fig. 3:**
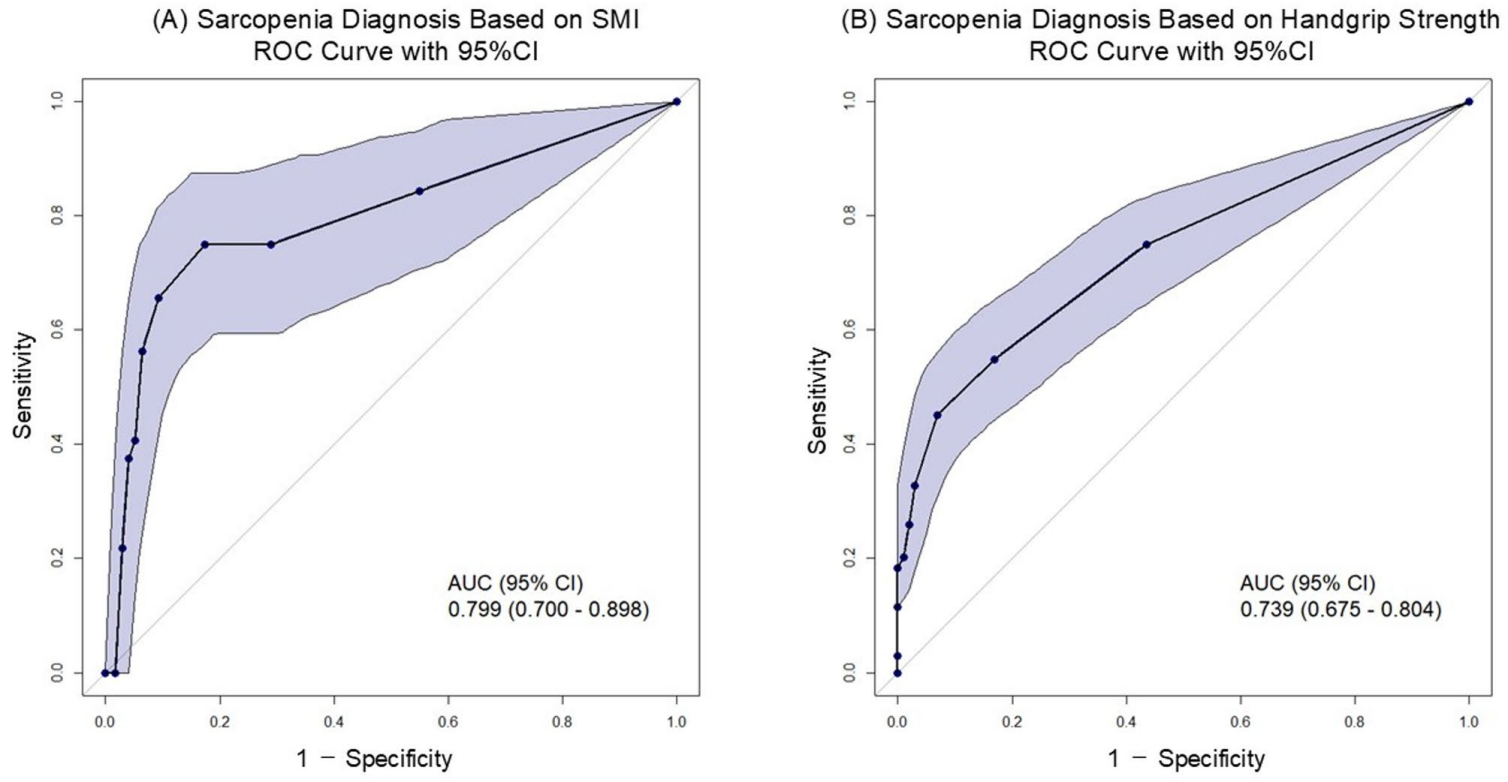
ROC curves of the SARC-F for sarcopenia diagnosis using two reference standards. (**A**) Diagnosis based on skeletal muscle index (SMI). (**B**) Diagnosis based on handgrip strength. Shaded areas represent 95% confidence intervals. The area under the curve (AUC) was 0.799 (95% CI: 0.700–0.898) for SMI-based diagnosis and 0.739 (95% CI: 0.675–0.804) for handgrip strength-based diagnosis.

Figure 3 shows the ROC curves evaluating the diagnostic performance of the SARC-F for identifying sarcopenia based on two reference standards: SMI derived from BIS (left panel) and handgrip strength (right panel). The AUCs for SARC-F in detecting sarcopenia defined by SMI and handgrip strength were 0.799 (95% CI: 0.700–0.898) and 0.739 (95% CI: 0.675–0.804), respectively.

## Discussion

We found the SARC-F score was significantly correlated with the electrical properties of tissues derived from BIS, such as PhA, ICW/ECW index, Cm, and fc, which are indices of muscle quality as well as muscle quantity^21,30^. SARC-F is a simple questionnaire consisting of five questions that can be easily incorporated as a screening tool in the clinic^7,8^. Previous studies have established a skeletal muscle composition model at the cellular level^20–22^. In this model, skeletal muscle contains not only functional muscle mass, but also ECW and intramuscular adipose tissue. DXA and traditional BIA are insufficient to explain the changes in muscle composition and muscle quality^21^. Functional muscle mass and muscle quality are essential biomarkers of muscle atrophy. Muscle power in the lower limbs is more closely associated with the MCM index, ICW/ECW index, PhA, Cm, and fc obtained by BIS than with the simple measures of appendicular lean mass obtained by DXA.

Previous studies suggested that the sensitivity of SARC-F is low, despite high specificity for sarcopenia screening with a score of  ≥ 4 points as the criterion. However, the SARC-F score had similar performance in predicting the incident physical limitation and mortality compared to actual sarcopenia definitions^10–14,49^. A recent study found that the SARC-F score was significantly correlated with the relative expansion of ECW against TBW (ECW/TBW) among 57 older male adults with gastrointestinal cancer^50^. Here, we demonstrated that the SARC-F score was correlated with both muscle quantity and quality in both men and women, independently of age, sex, height, and BMI. Thus, SARC-F itself may be useful for screening of actual muscle atrophy and/or loss of muscle quality.

As a limitation, our study design was cross-sectional research, and further longitudinal research are needed. Another limitation of this study is that although individuals with apparent cognitive impairment were excluded based on medical records, the full spectrum of neuromotor and cognitive impairments was not comprehensively assessed. Undiagnosed mild cognitive impairment could not be entirely ruled out, which may have influenced the accuracy of responses to the SARC-F, as it includes subjective components. Additionally, a small number of participants had a history of stroke, potentially affecting motor function and SARC-F scores^51^. Due to the limited sample size and variability in impairment severity, detailed analyses were not possible. Future studies should incorporate standardized assessments of both neuromotor and cognitive functions to better control for these potential confounding factors. Future research will also be required to evaluate SARC-F by family members or caregivers who are able to check daily situations.

As the Global Leadership Initiative for Sarcopenia pointed out there were different definitions of muscle quality^52^. In our results, we found the SARC-F was associated with various aspects of muscle quality derived from BIS parameters. A previous study has shown that muscle mass and quality are independent of muscle strength^53^. Future research is expected to deliver new methods for evaluating skeletal muscle mass and quality that integrate these variables. Additionally, as a combined public health and clinical approach, a two-tiered assessment using SARC-F and BIS may help clinicians better understand the skeletal muscle status of their subjects.

An important point of our finding is that SARC-F was associated with muscle quality evaluated by BIS. PhA, one of the widely used muscle assessments, has been remarked upon because it is able to predict mortality and various health outcomes^54–56^. In our results, the association between SARC-F and PhA was moderate. Lusaki reported a review which showed bioelectrical impedance vector analysis (BIVA) assists in understanding the result. BIVA showed that “cachexic” individuals had lower PhA, while “fit and muscular” and “obese normo-hydrated” had higher PhA than “normal”, respectively^57^. It might explain the moderate association in our result, as it is able to understand that PhA in athletes and obese individuals tends to be similar.

In conclusion, the current study indicates that the SARC-F score is associated with electrical properties of tissues assessed by BIS including PhA and ICW/ECW index, independent of other variables in older adults and emphasizes the usefulness of SARC-F as an assessment tool for actual sarcopenia (i.e. muscle atrophy and loss of muscle quality).

## Data Availability

The datasets generated and/or analyzed during the current study are not publicly available due to ethical restrictions, but they are available from the corresponding author upon reasonable request.
